# Mode of action of antimicrobial agents albofungins in eradicating penicillin- and cephalosporin-resistant *Vibrio parahaemolyticus* biofilm

**DOI:** 10.1128/spectrum.01563-23

**Published:** 2023-08-23

**Authors:** Weiyi She, Aifang Cheng, Wenkang Ye, Ping Zeng, Hao Wang, Pei-Yuan Qian

**Affiliations:** 1 Southern Marine Science and Engineering Guangdong Laboratory (Guangzhou), Guangdong, China; 2 Department of Ocean Science, Hong Kong University of Science and Technology, Hong Kong, China; 3 School of Pharmacy, Faculty of Medicine, The Chinese University of Hong Kong, Hong Kong, China; Shenzhen Bay Laboratory, Cambridge, Massachusetts, USA

**Keywords:** albofungin, drug-resistant strain, membrane damage, proteomics, *Vibrio parahaemolyticus*

## Abstract

Albofungin is a promising broad-spectrum antimicrobial compound against multidrug-resistant bacteria. In the present study, we further investigated albofungin’s biofilm eradication activity and its potential mode of action against drug-resistant *Vibrio parahaemolyticus*. Among all derivatives, albofungin exhibited the best antibiofilm and antibacterial activity with rapid killing effects at 0.12 µg mL^−1^. Confocal microscopy observation exhibited that albofungin disrupted *V. parahaemolyticus* biofilms by killing or dispersing biofilm cells. Meanwhile, scanning electron microscope and fluorescent staining experiments demonstrated that albofungin rapidly destroyed the integrity and permeability of the bacterial cell membrane. Moreover, this study revealed an antibiofilm mechanism of albofungin involving inhibition of peptidoglycan biosynthesis, flagella assembly pathways, and secretion system proteins in *V. parahaemolyticus* by quantitative proteomics and validation experiments. Our results highlighted albofungin’s mechanism of action in planktonic cells and biofilms and suggested further development and potential applications of albofungin for treating infections caused by penicillins-and-cephalosporins-resistant *V. parahaemolyticus*.

**IMPORTANCE:**

Infections caused by multidrug-resistant bacteria, as well as a scarcity of new antibiotics, have become a major health threat worldwide. To tackle the demand for new and effective treatments, we investigated the mechanism of action of albofungin, a natural product derived from *Streptomyces*, which exhibits potent antimicrobial activity against multidrug-resistant bacteria. Albofungin showed potent biofilm eradication activity against penicillins-and-cephalosporins-resistant *Vibrio parahaemolyticus*, which expresses a novel metallo-β-lactamase and, thus, reduces their sensitivity to various antibiotics. We observed membrane disruption and permeation mechanisms in planktonic cells and biofilms after albofungin treatment, while albofungin had a weak interaction with bacterial DNA. Moreover, the antibiofilm mechanism of albofungin included inhibition of peptidoglycan biosynthesis, flagellar assembly pathways, and secretion system proteins. Our finding suggested potential applications of albofungin as an antibacterial and antibiofilm therapeutic agent.

## INTRODUCTION

The rise of multidrug-resistant bacteria led to a significant threat to global human health (1). These pathogenic microorganisms are capable of forming biofilms on various surfaces, such as medical equipment, environmental objects, and food packaging materials (2). Biofilms possess a remarkable 10- to 1,000-fold greater resistance to antimicrobial compounds, compared to planktonic cells, largely due to the presence of extracellular polymeric substances (EPSs) that prevent penetration into the matrix (3, 4).

*Vibrio parahaemolyticus* is a Gram-negative pathogen found in estuarine, marine, and coastal environments that cause food-borne disease (5, 6). *V. parahaemolyticus* can survive in various environmental conditions, thereby forming biofilms on the surface of materials such as plastic, glass, stainless steel, and polyurethane (7). The most common infections caused by *V. parahaemolyticus* are gastroenteritis, wound infections, ear infections, and septicemia (8, 9). In addition, vibriosis obstructs aquaculture development, which imposes a huge cost on the aquaculture industry annually (10, 11). During the past couple of decades, abuse of antibiotics has greatly contributed to antimicrobial resistance spread among *Vibrio* spp. (10). For example, *V. parahaemolyticus* isolated from shellfish and shrimp develops resistance against various antibiotics such as ampicillin, chloramphenicol, tetracycline, levofloxacin, kanamycin, and gentamicin (12, 13).

Due to the high prevalence of drug-resistant *Vibrio* strains and their ability to form biofilm, it is imperative to develop novel and potent antibiofilm agents to effectively combat *Vibrio*-biofilm infections (14). However, eradicating biofilms using conventional antibiotics is extremely challenging; therefore, exploring the antibacterial and antibiofilm properties of active compounds derived from natural products presents a promising alternative to the development of new antibiofilm agents. As a polycyclic isoquinolone-xanthone compound first isolated from *Actinomyces* in the 1970s (15, 16) and later discovered in different *Streptomyces* species (17), albofungin shows a wide range of potent bioactivities, including antitumor, antifungal, and anti-HIV activity (17 – 19). More importantly, albofungin exhibits predominant antibacterial and antibiofilm properties according to our previous studies, which can be utilized to reduce biofouling in marine environments (18, 20). For example, albofungin was capable of inhibiting the formation of biofilms in multiple strains of marine bacteria and “ESKAPE pathogens,” including *Staphylococcus aureus*, *Sulfitobacter pontiacus*, *Escherichia coli*, and *Acinetobacter baumannii* with low cytotoxicity (18, 20). Furthermore, albofungin binds to the transglycosylase domain of penicillin-binding proteins in cell wall biosynthesis (21). However, how it works to combat bacteria and biofilms remains elusive. An understanding of the mechanism of action of a new antimicrobial agent, albofungin, is essential when evaluating it for clinical applications so that potential toxic effects can be predicted and the structure of albofungin can be redesigned to enhance its antibiofilm properties.

In the present study, we further explored albofungin’s antibacterial and antibiofilm mode of action against drug-resistant *V. parahaemolyticus*, which expresses a novel metallo-β-lactamase and, thus, reduces their sensitivity to various antibiotics. Intriguingly, the in-depth proteomics analysis also highlighted that albofungin particularly inhibited the key biofilm formation proteins in the *V. parahaemolyticus*, which provided insights into its antibiofilm mode of action. Overall, our findings suggested albofungin’s potential applications to multidrug-resistant Gram-negative pathogens.

## RESULTS

### Isolation of albofungin derivatives and their activities against drug-resistant *Vibrio* strains

**Fig 1:**
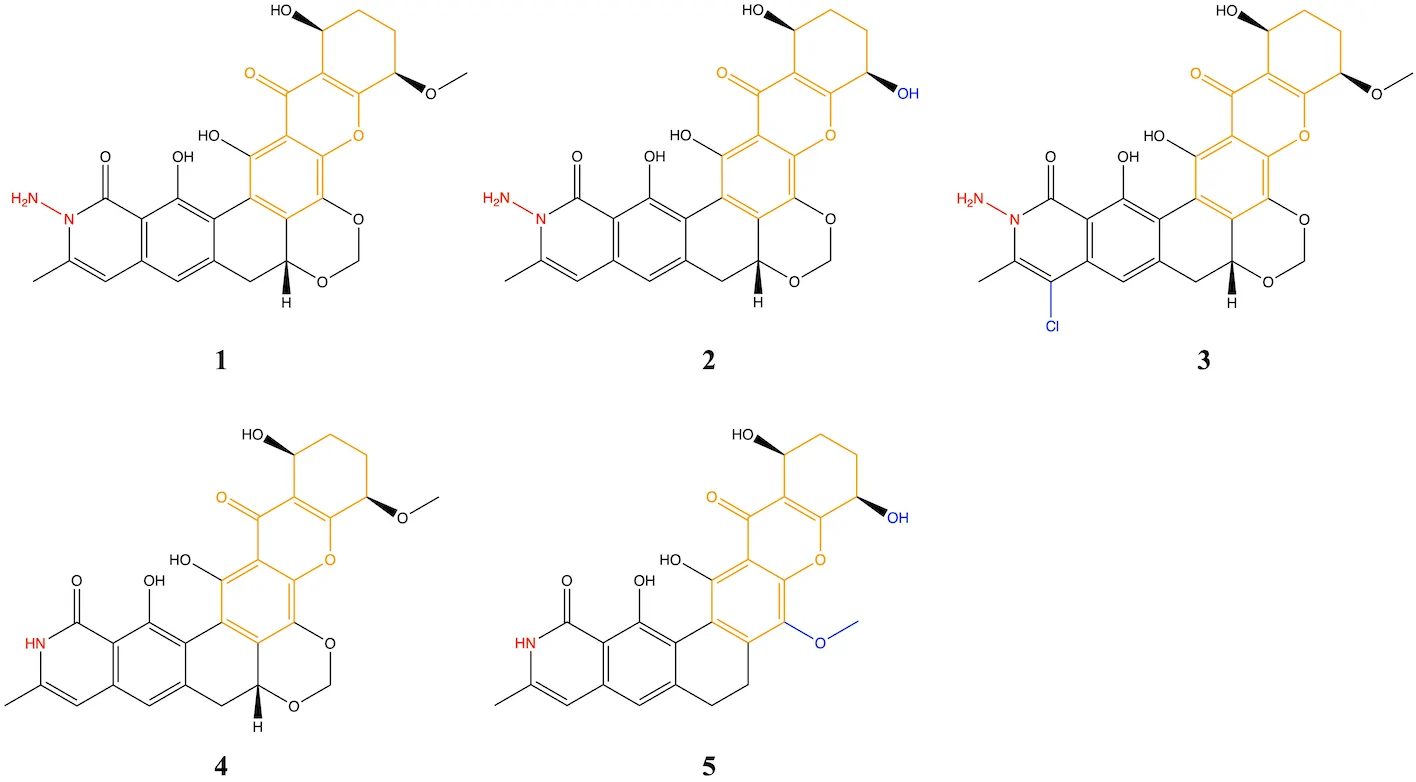
Chemical structures of albofungins isolated from *Streptomyces chrestomyceticus* BCC 24770.

**Fig 2:**
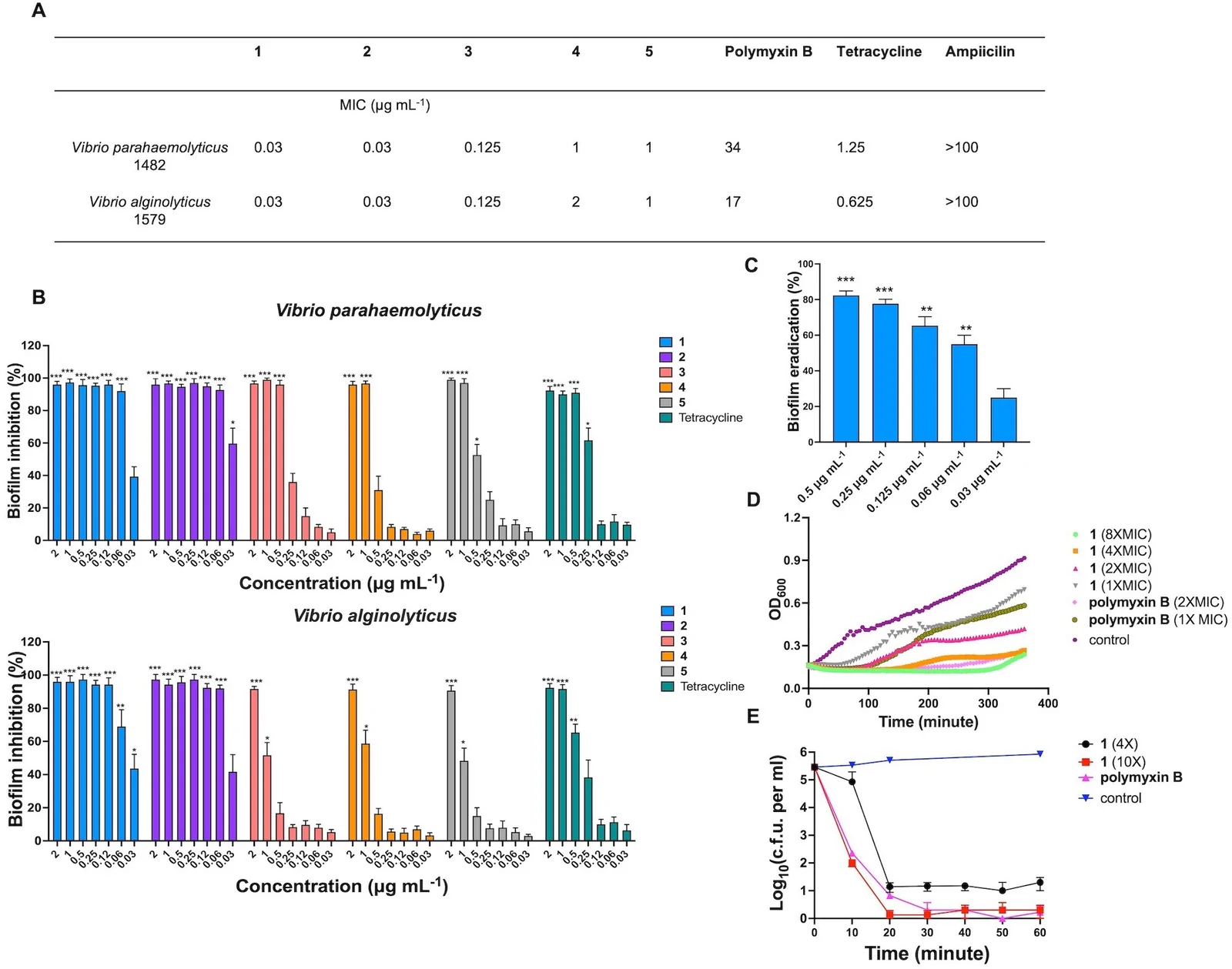
Antibacterial and antibiofilm study of *Vibrio parahaemolyticus* 1482 and *Vibrio alginolyticus* 1579 upon albofungins (1 – 5) treatment. (**A**) MIC values of albofungins (1–5) to *Vibrio* strains. (**B**) Inhibition of biofilm formation by albofungins (1–5) against *V. parahaemolyticus* 1482 and *V. alginolyticus* 1579. Error bars represent SD, *n* = 9 wells from three batches of microbial cultures. Significant differences were analyzed by one-way ANOVA compared with the control biofilm, *P < 0.05, **P < 0.01, and ***P < 0.001. (**C**) Eradication of biofilm formation by albofungin against *V. parahaemolyticus* 1482. Error bars represent SD, *n* = 9 wells from three batches of microbial cultures. Significant differences were analyzed by one-way ANOVA compared with the control biofilm, *P < 0.05, **P < 0.01, and ***P < 0.001. (**D**) Growth curves and (**E**) time-kill curves of *V. parahaemolyticus* 1482 after albofungin treatment. Error bars represent SD.

Five compounds (1–5) were isolated from *Streptomyces chrestomyceticus* BCC 24770, including albofungin (1), albofungin A (2), chloroalbofungin (3), chrestoxanthone A (4), and chrestoxanthone C (5) (Fig. 1; Fig. S1). The antimicrobial potency of compounds 1–5 against drug-resistant *Vibrio* strains was assessed, revealing MIC values within the range of 0.03–2 μg mL^−1^. However, ampicillin showed no activity up to 100 µg mL^−1^, and polymyxin B also had moderate activity. Albofungin exhibited the strongest antibacterial activity (Fig. 2A) and relatively low hemolytic activity), and thus, it was selected for further study.

Given that *V. parahaemolyticus* is a well-known pathogen that is capable of forming biofilms, the compounds were tested for their biofilm formation (inhibition effect) and biofilm clearance (eradication effect) activities. All compounds exhibited biofilm inhibition activity against *V. parahaemolyticus* 1482 and *V. alginolyticus* 1597*,* as shown in Fig. 2B. Albofungin (1) and albofungin A (2) showed the most potent biofilm inhibition activities which inhibited 90% of biofilms at 2 × MIC, and their MBIC_90_ value was nearly 10 times as compared with that of the tetracycline. At 1 × MIC value, albofungin (1) and albofungin A (2) reduced 39% and 59% of biofilm biomasses, respectively, as compared with the control. Furthermore, albofungin could eradicate 50% of the biofilms at 0.06 µg mL^−1^ (Fig. 2C).

### Time-kill and growth kinetics of *Vibrio* upon albofungin treatment

The growth curve showed that albofungin remarkably inhibited the proliferation of *V. parahaemolyticus* 1,482 in a dose-dependent manner, and its growth was completely inhibited with 4 × MIC (Fig. 2D). Thus, time-kill assays at 4 × MIC and 10 × MIC values were further performed and confirmed that a high concentration of albofungin exhibited a bactericidal mode of action. Albofungin killed more than 99.9% of *Vibrio* cells at 4 × MIC and 10 × MIC values within 20 min, and the quickness of the killing effect was similar to polymyxin B, as shown in Fig. 2E.

### Proteomic analysis of planktonic bacteria and biofilm upon albofungin treatment

**Fig 3:**
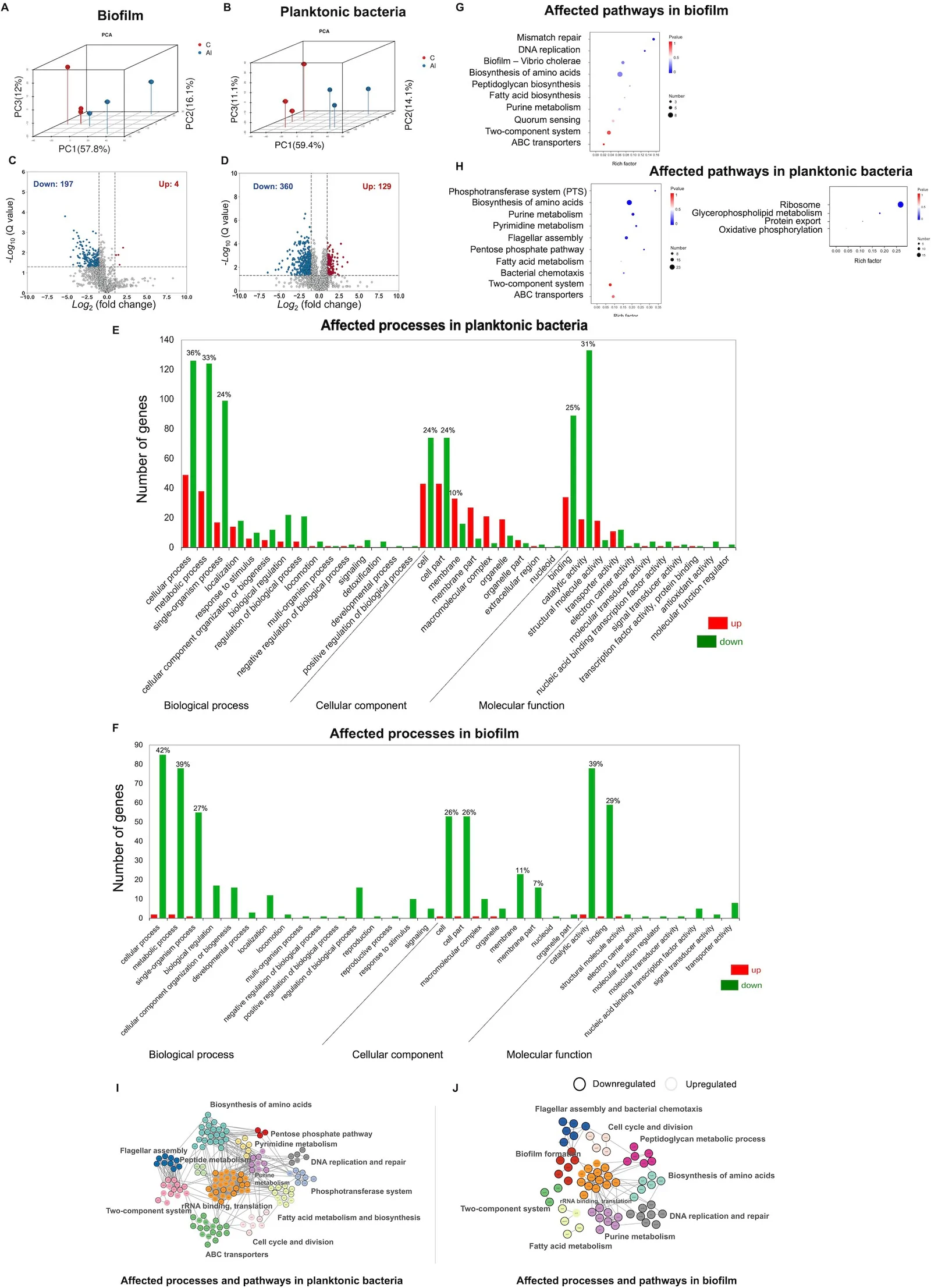
Proteomics analysis of planktonic bacteria and biofilm upon albofungin treatment. (**A and B**) Principal-component analysis (PCA) of biofilm (**A**) and planktonic bacteria (**B**) after albofungin treatment. (**C and D**) Volcano plots of the biofilm (**C**) and planktonic bacteria (**D**) after albofungin treatment. (**E and F**) Gene ontology (GO) enrichment analysis of the affected processes in planktonic bacteria (**E**) and (**F**) biofilm after albofungin treatment. (**G and H**) Kyoto Encyclopedia of Genes and Genomes (KEGG) enrichment analysis of the affected pathways in (**G**) biofilm and (**H**) planktonic bacteria after albofungin treatment. (**I and J**) Protein-protein interaction network of the DEPs between the planktonic bacteria (**I**) and biofilm (**J**), as predicted by STRING v11.0 and visualized by Cytoscape. Lines and dots in different colors represent protein interaction and different protein functions, respectively.

A label-free quantitative proteomics was performed on the planktonic bacteria and biofilm samples (with or without albofungin treatment) to have a deeper understanding of the affected biological processes by albofungin. We observed from the principal component analysis (PCA) plots that the albofungin-treated and control groups were well separated from each other, and there also had a clear difference between the planktonic bacteria control and biofilm control samples (Fig. 3A and B; Fig. S3). The volcano plots indicated that there was a total of 489 differentially expressed proteins (DEPs) in the albofungin-treated logarithmic-phase planktonic bacteria group and 201 DEPs in the albofungin-treated biofilm group as compared with the control group (Fig. 3C and D), which suggested that the biofilms were more resistant than planktonic bacteria to albofungin. From GO enrichment analysis, more than 30% of proteins in the cellular, metabolic, and catalytic activity processes were significantly changed upon albofungin treatment (Fig. 3E and F; Fig. S4), thereby showing that albofungin may inhibit the proliferation of *V. parahaemolyticus*. More than 10% of membrane-related proteins were significantly changed, which implied that the cell membrane was targeted when applied with albofungin. Biosynthesis of amino acid, purine metabolism, and fatty acid biosynthesis were significantly downregulated in both treatment groups, as revealed by KEGG enrichment analysis (Fig. 3G and H).

**Fig 4:**
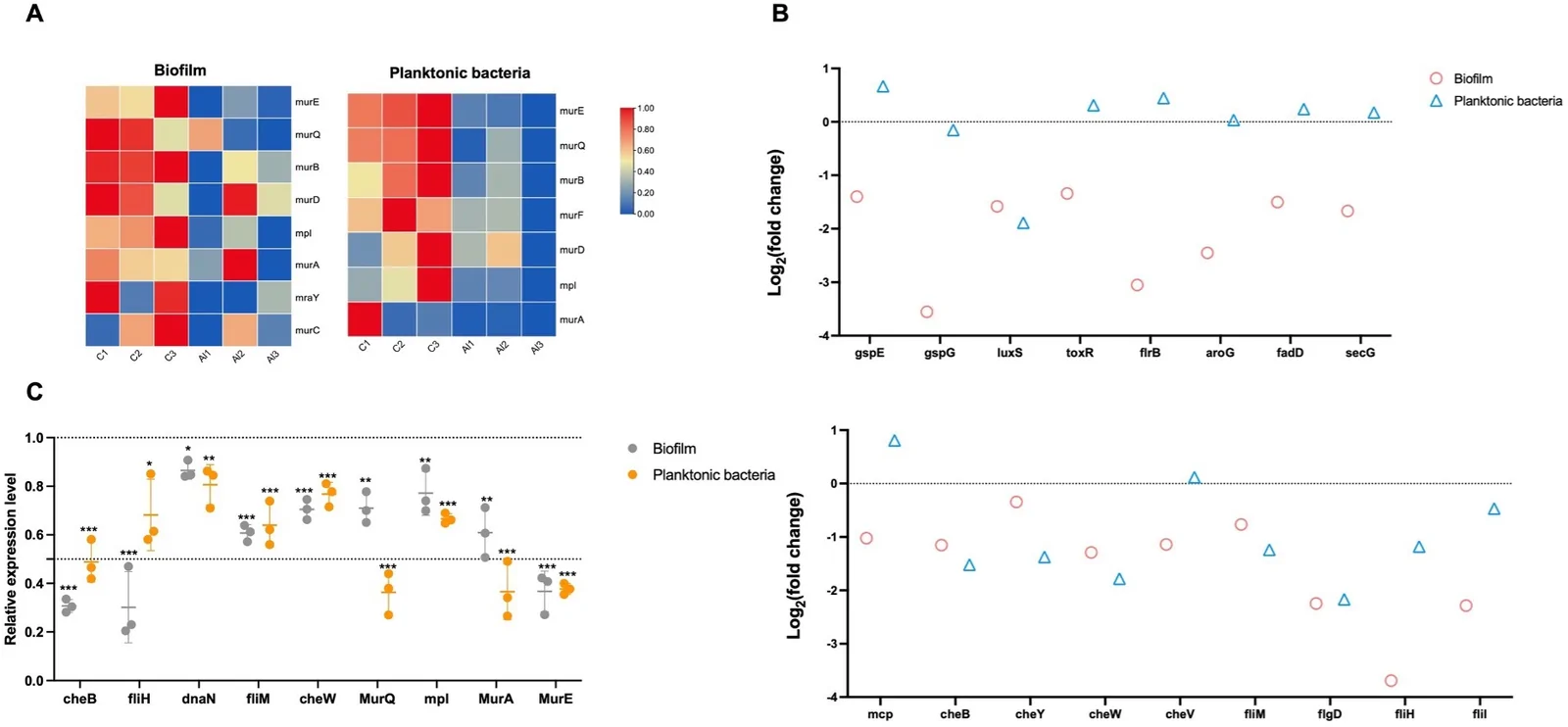
Expression pattern of selected processes in the planktonic bacteria and biofilm upon albofungin treatment. (**A**) Heat map of the expression level of selected proteins in the peptidoglycan biosynthesis pathway. (**B**) Log_2_ (fold change) of selected proteins in the biofilm formation pathway (upper) and bacterial chemotaxis and flagellar assembly pathways (lower). (**C**) qPCR analysis of nine genes in the selected pathways. Error bars denote SD. Significant differences were analyzed by one-way ANOVA, *P < 0.05, **P < 0.01, and ***P < 0.001.

Peptidoglycan is a rigid envelope surrounding the cytoplasmic membrane, which plays an important role in bacterial structural support and protection (22). We observed that proteins such as mpl, murE, mraY, murQ, and murB that participated in the peptidoglycan biosynthesis were significantly reduced (>twofold) in the albofungin-treated biofilm group as compared with the control group (Fig. 4A). Others such as murA, murC, and murD were downregulated in the biofilm and planktonic bacteria groups after albofungin treatment, indicating that the peptidoglycan biosynthesis pathway was inhibited in the presence of albofungin (Fig. 4A). In addition, proteins necessary for DNA replication and repair [such as replicative DNA helicase (dnaB), DNA polymerase III subunit beta (dnaN), and DNA polymerase III subunit epsilon (dnaQ)] were significantly downregulated after albofungin treatment. Based on the results of the DNA interaction assay, albofungin showed to bind with DNA *in vitro* at a 1:16 ratio (Fig. S5); hence, we suggested that the DNA replication and mismatch repair process in the *V. parahaemolyticus* cells were impaired after albofungin treatment.

Interestingly, some secretion system proteins (e.g., gspE and gspG) and toxin transcriptional activators (toxR) were significantly downregulated in the biofilm samples, whereas no significant changes were observed in the planktonic bacteria samples after alboufngin treatment (Fig. 4B), which may indicate the potential targets of albofungin in biofilms. Flagella-mediated motility promotes the early stages of *Vibrio* biofilm formation, and proteins related to flagellar assembly (fliH, fliD, and fliM) and bacterial chemotaxis (cheB, cheV, and mcp) were also downregulated in albofungin-treated groups, which suggested that albofungin inhibited *V. parahaemolyticus* biofilm formation and development.

Therefore, some genes involved in the peptidoglycan biosynthesis, bacterial chemotaxis, and flagellar assembly pathways were selected to analyze their relative expression levels to validate the proteomics results. Consequently, all the genes were downregulated in the albofungin-treated planktonic bacteria and biofilm groups (Fig. 4C). In particular, MurE was significantly downregulated in the biofilm samples (0.37-fold) and planktonic bacterial samples (0.38-fold) after albofungin treatment as compared with the control group. MurA and MurQ were downregulated in the planktonic bacterial samples (0.37- and 0.36-fold, respectively), whereas fliH and cheB were downregulated in the biofilm samples (0.30- and 0.31-fold, respectively) after albofungin treatment as compared with the control group.

### SEM and CLSM analyses of biofilm structures in response to albofungin

**Fig 5:**
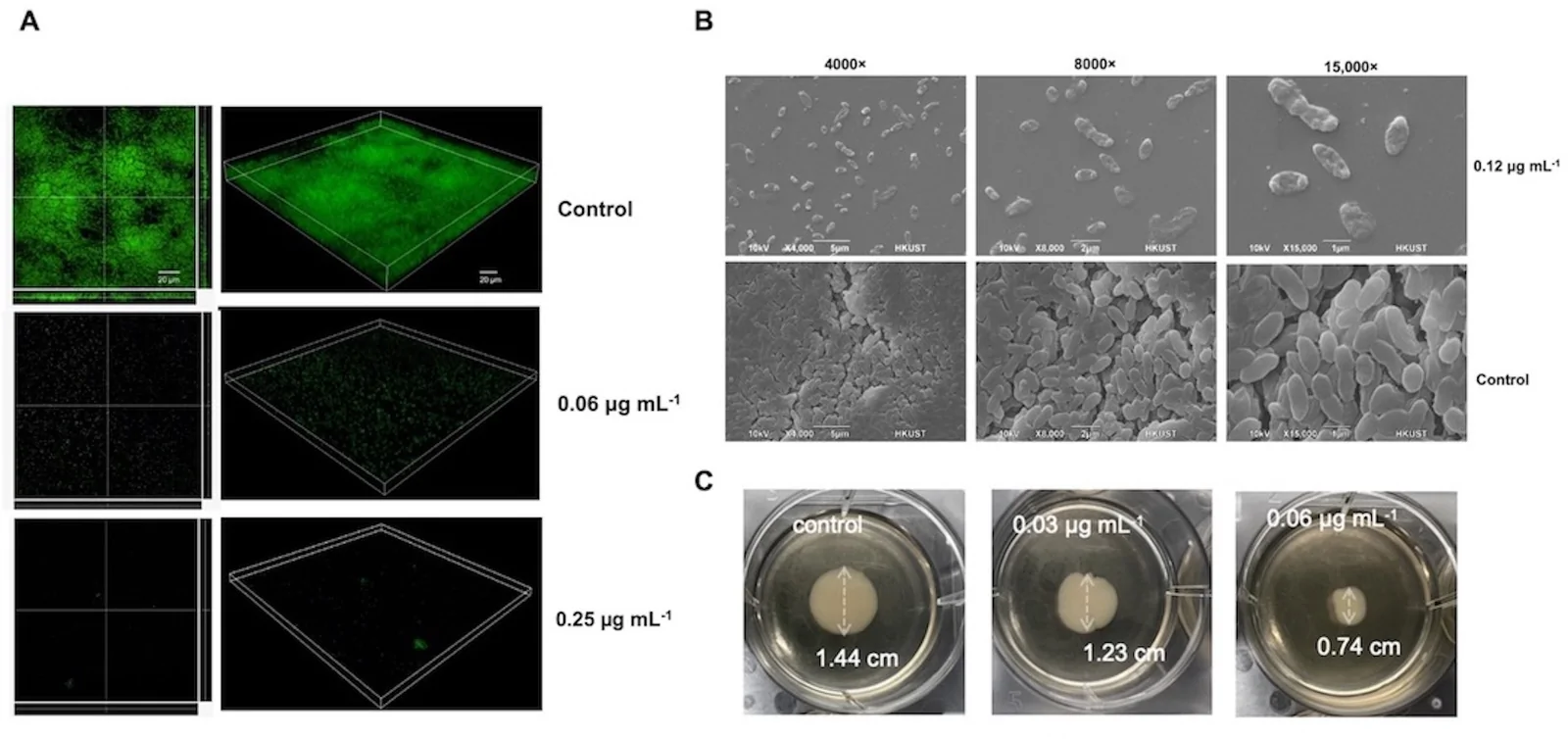
Antibiofilm activity of albofungin against *Vibrio parahaemolyticus*. (**A**) Confocal microscopy of preformed biofilm (20 h) treated with different concentrations of albofungin. (**B**) SEM images of preformed biofilm treated with 0.12 µg mL^−1^ albofungin. (**C**) Swarming motility test of 1 to *V. parahaemolyticus*.

The 24-h preformed mature biofilm structures after being treated with albofungin were visualized with scanning electron microscopy (SEM) and confocal laser scanning microscopy (CLSM). A 3D image of *V. parahaemolyticus* biofilms revealed the thickness and fluorescence intensity of bacteria cells within it (Fig. 5A). Figure 5A showed that 0.06 and 0.25 µg mL^−1^ of albofungin were highly effective in removing viable cells, and the effects were proportional to their concentrations, which confirmed the bioassay results. The SEM images showed that the biofilm mass was reduced and cell morphology greatly changed with serious deformation after 0.12 µg mL^−1^ albofungin treatment (Fig. 5B). Swarming motility is critical for attachment to surfaces in the early stages of biofilm formation (23). The swarming diameter decreased to 1.23 and 0.74 cm in albofungin-treated groups as compared to untreated group (Fig. 5C), which suggested that albofungin could control the spread of this pathogenic bacteria and, therefore, inhibit the biofilm formation.

### Albofungins affected the membrane of *Vibrio* cells

**Fig 6:**
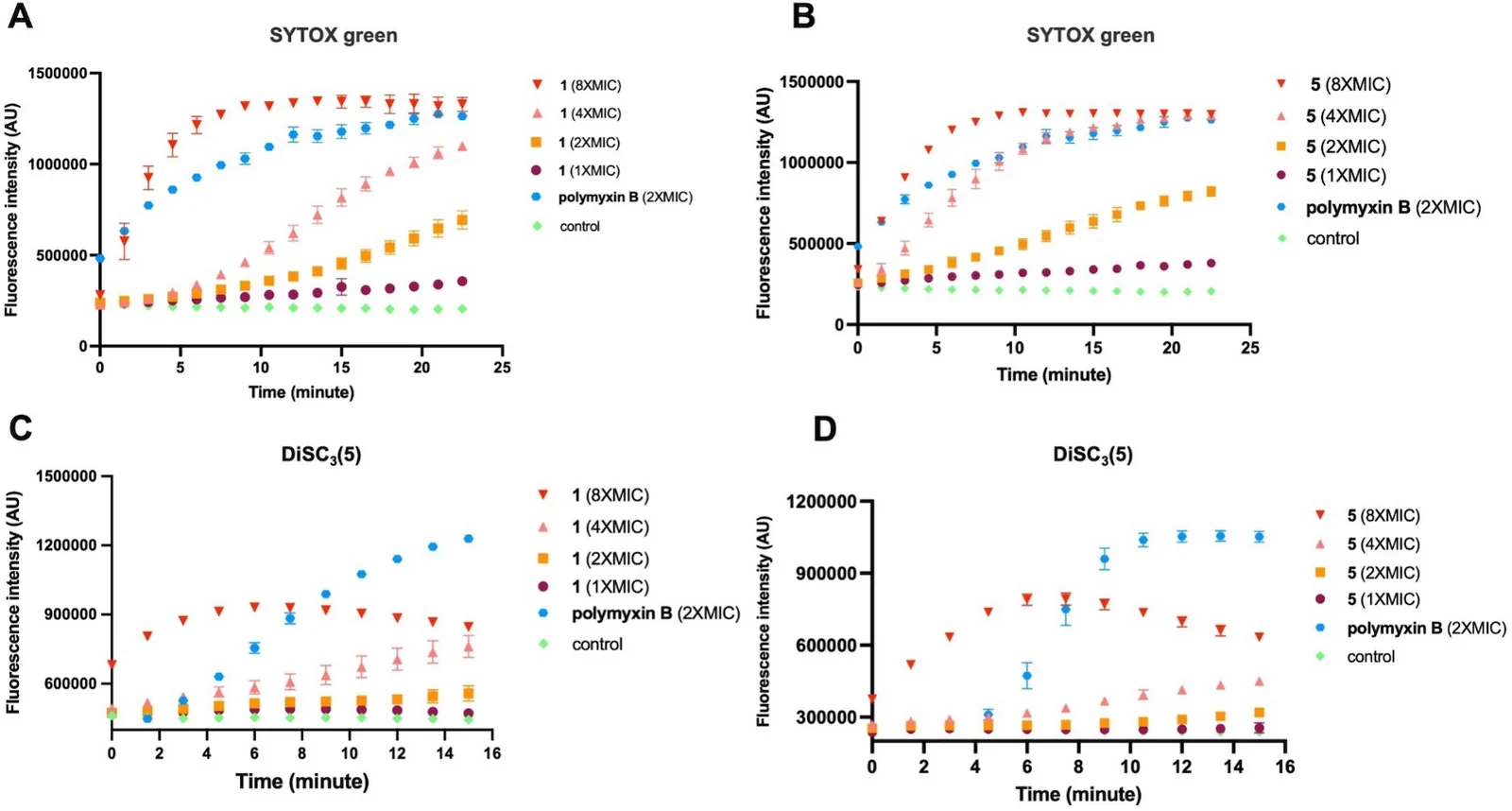
Membrane alteration of *Vibrio parahaemolyticus* after treatment of albofungin (1) and chrestoxanthone C (5). Membrane integrity of *V. parahaemolyticus* after (**A**) albofungin (1) and (**B**) chrestoxanthone C treatment (5) was evaluated by SYTOX green. Depolarization of the membrane of *V. parahaemolyticus* after treatment with (**C**) albofungin (1) and (**D**) chrestoxanthone C (5) was evaluated by DiSC_3_(5).

The membrane integrity of *V. parahaemolyticus* cells after albofungin treatment was evaluated using the SYTOX Green dye. SYTOX Green, which is bound to the nucleic acids, could enter the damaged cells but is impermeant to live cells. As shown in Fig. 6A and B, upon albofungin (1) or chrestoxanthone C (5) treatment, the fluorescence intensity of SYTOX Green rapidly increased within 25 min, which was positively correlated with the compound concentrations. Moreover, the albofungin-induced membrane potential was evaluated by DiSC_3_(5) dye, a membrane potential sensitive probe, which was accumulated in the cytoplasmic membrane of bacteria; when the membrane is depolarized, the dye will be released. The fluorescence intensities of DiSC_3_(5) increased when the concentrations of albofungin (1) and chrestoxanthone C (5) increased (Fig. 6C and D). However, the increase of fluorescence intensity in the treatment of 2 × MIC of polymyxin B was much higher than in all albofungin-treated groups, which suggested that albofungins were not efficient membrane dissipators. Furthermore, the interaction between LPS and albofungin was determined based on the isothermal titration calorimetry (ITC) experiment; however, no binding affinity was observed (Fig. S6), indicating that the bacterial outer membrane permeability was not influenced by albofungin. Therefore, our results suggested that albofungin destroyed the membrane by making pores in the cytoplasmic membrane.

### Cell morphology changes upon albofungin treatment

**Fig 7:**
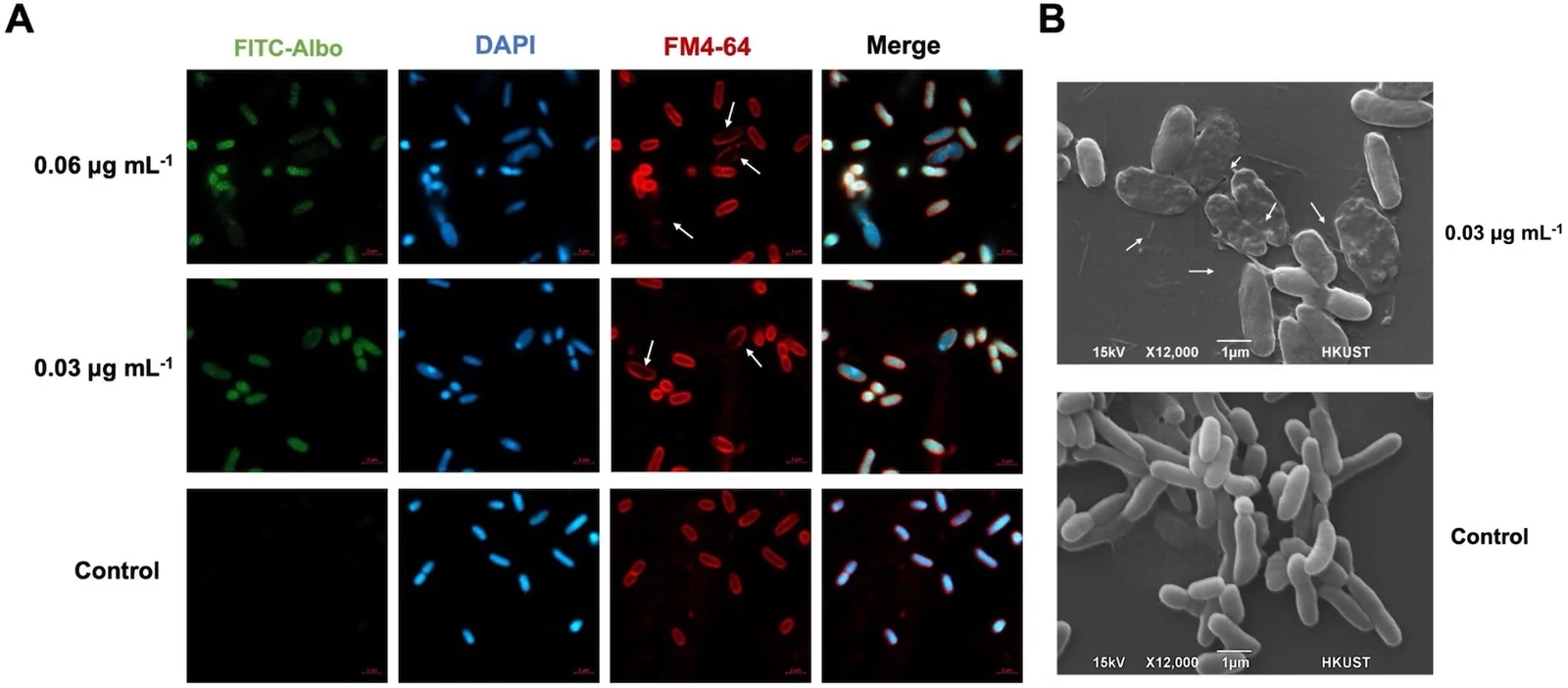
Fluorescence and electron microscopy. (**A**) Fluorescence microscopy of *Vibrio parahaemolyticus* after FITC-albofungin treatment and stained with FM4-64, DAPI. (**B**) SEM images of *V. parahaemolyticus* after albofungin treatment. White arrows denote membrane deformation and detachment.

To determine the antibacterial and antibiofilm mode of actions of albofungin, we used confocal imaging to examine the localization of albofungin in the *V. parahaemolyticus* cells and observed after staining with membrane dye FM4-64, nucleic acid stain DAPI, and FITC-albofungin. The blue fluorescence signal of DAPI was observed uniformly in all the tested groups, whereas only the albofungin-treated groups exhibited green fluorescence signals, which overlapped well with the blue fluorescence signals in the merged images. When stained with FM4-64, the red fluorescence signals were detected clearly in the cell membrane, whereas weaker or even no membrane signals were observed in treated cells (indicated in the white arrows, Fig. 7A). Results indicated that the FITC-albofungin quickly penetrated the cell membrane, accumulated in the cytoplasm and nucleus, and the membrane structures were destroyed by albofungin with cytoplasmic leakage. This point was further clarified through SEM observation. Cells exhibited a clubbed morphology with a smooth surface and intact outline under normal conditions (Fig. 7B). However, morphological changes in cells were observed after albofungin treatment, wherein some of the cells were deformed, and they became flat, rough, and inflated.

## DISCUSSION

Albofungin exhibited broad-spectrum antibacterial and antibiofilm activities against Gram-negative and Gram-positive bacteria (18, 20). It can be used as a coating agent in marine environments because of its significant antifouling activity and low cytotoxicity (20). The present study is the first to elucidate the possible underlying mode of action of albofungin against Gram-negative bacteria. The superbugs *V. parahaemolyticus* and *V. alginolyticus* are biofilm-forming pathogens, which trigger human acute gastroenteritis and diarrhea, thus albofungin also shows great potential and research significance in medical biofilm eradication for clinical setup (24, 25). Herein, these two strains in the present study were isolated from seafood in a previous study that expressed novel metallo-β-lactamases, which decrease their sensitivity to various antibiotics including ceftazidime (CAZ), penicillin G (PenG), amoxicillin (AMX), oxacillin (OXA), ampicillin (AMP), and so on (26, 27). Interestingly, these *Vibrio* strains showed sensitivity to albofungins, particularly albofungin and albofungin A, with a MIC value of 0.03 µg mL^−1^.

The time-kill assay suggested that albofungin influenced bacterial growth in a bactericidal mode. Polymyxin B was used as the positive control because it was effective for various Gram-negative infections by damaging the cytoplasmic membrane (28) and showed quick bactericidal kinetics unlike some other antibiotics that primarily target intracellular components and have slow-killing kinetics (29). Similarly, albofungin showed a quick-kill effect equivalent to polymyxin B, thereby eliminating 99% of bacteria in a short period. Along with the SYTOX green staining and SEM experiment, albofungin’s antibacterial effect worked by membrane permeation and disruption; therefore, resistance was less likely to develop. In addition, albofungin has an -NH_2_ group which may possess positively charged properties in water solution. However, it showed no binding affinity with LPS, a critical component of the outer membrane of the Gram-negative bacteria with a negatively charged site (30). Thus, our results may indicate that albofungin’s antibacterial mode of action was not similar to the cationic antibacterial peptides such as polymyxin B.

The use of mass spectrometry-based quantitative proteomics gives us a good understanding of the bacterial response to extraneous substances (31). Herein, we described for the first time the proteomic profiles of *V. parahaemolyticus* under albofungin treatment. As the most significant finding, various proteins were downregulated in the biofilm and planktonic bacteria groups, and there were more variations of protein expressions in planktonic bacteria than in biofilms after albofungin treatment, which indicated that biofilms were more resistant to extraneous compounds than planktonic bacteria. Moreover, various metabolic processes were influenced by albofungin including the most significantly downregulated pathways, such as purine metabolism (purN, purK, and hpt), biosynthesis of amino acids (hisF, hisH, and dapE), and fatty acid metabolism (fabD, fabB, and fabG). As an inhibitor of penicillin-binding proteins, the presence of albofungin accumulated the cell wall precursor UDP-MurNAc pentapeptide and targeted the peptidoglycan biosynthesis pathway (21). We were interested in how albofungin affects peptidoglycan biosynthesis and regulates the proteins that participated in this pathway. As we observed, most of the proteins involved in the peptidoglycan biosynthesis pathway (mpl, murE, mraY, murQ, murB, murA, murC, and murD) were significantly downregulated after albofungin treatment, thereby indicating that the formation of the rigid envelope was damaged, and exogenous compounds could enter the cytoplasmic membrane easily.

However, biofilm infections are difficult to eradicate with antimicrobial agents as compared to planktonic cells because biofilms are highly resistant (32, 33). Albofungin and albofungin A exhibited strong biofilm inhibition activities (inhibition of 90% biofilm) at 2 × MIC. In addition to interfering with the survival of planktonic bacteria, we believed that albofungin also affected some proteins involved in the processes of biofilm formation. The formation of biofilms is influenced by various genetic factors, including quorum sensing, flagella, pili, exopolysaccharide, c-di-GMP signaling, and two-component regulators (34, 35). Flagella triggers the initial stages of biofilm formation by stimulating movement toward and along the surface (36). Our proteomics and qPCR analysis showed that proteins related to flagella assembly and bacterial chemotaxis, including fliM, fliH, cheB, and cheW, were significantly downregulated. The biofilm inhibition effects of albofungin were further confirmed by the experiment of swarming motility driven by rotating flagella. Some secretion system proteins (gspE and gspG) and toxin transcriptional activators (toxR) were highly expressed in the biofilms, but they were significantly downregulated in the albofungin-treated biofilms as compared with albofungin-treated planktonic bacteria. The results indicated the overall inhibition of biofilm formation by albofungin.

**Fig 8:**
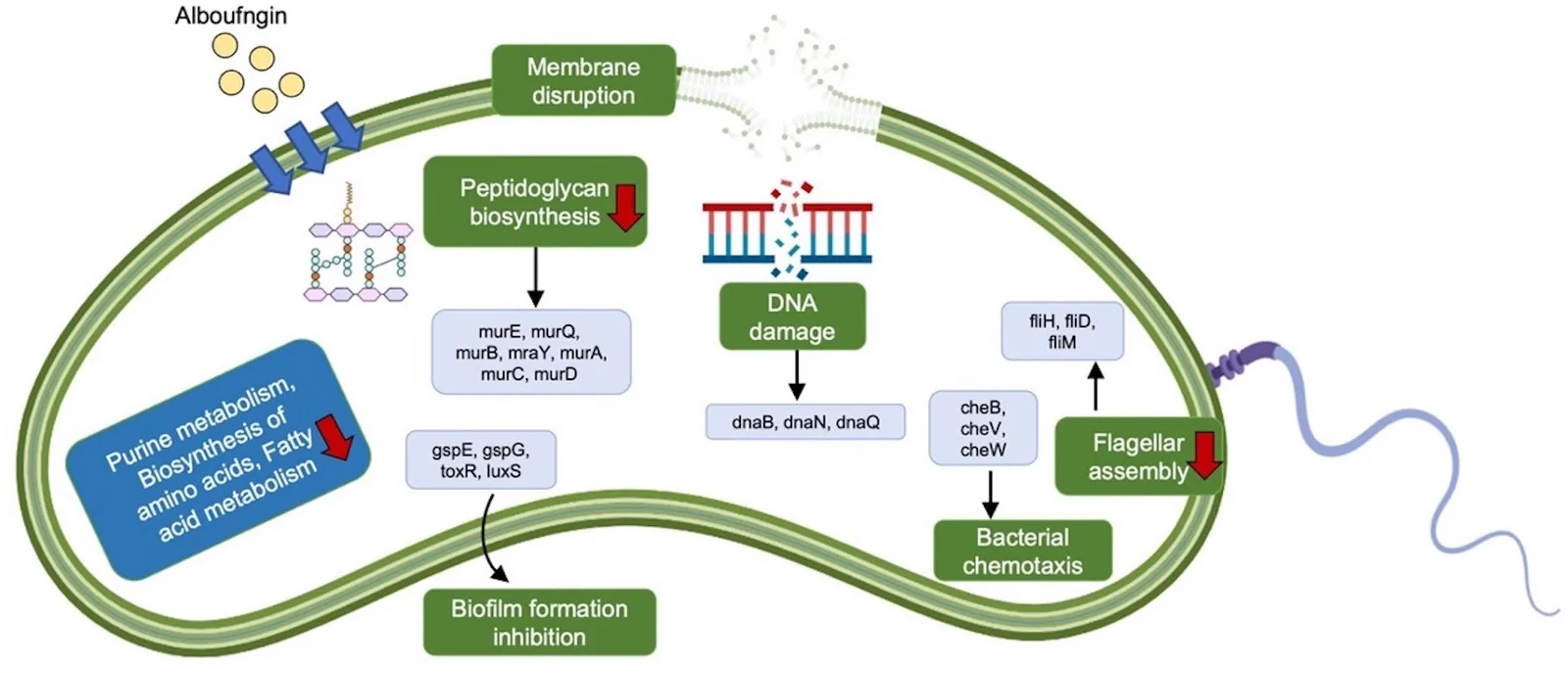
Proposed mode of action of albofungin in *Vibrio parahaemolyticus*.

In summary, our study demonstrated that albofungin killed the planktonic bacteria, as well as effectively inhibited and eradicated the preformed biofilms of drug-resistant *Vibrio* strains. Albofungin’s mechanisms of action involved rapidly disrupting and permeabilizing the bacterial membrane, followed by interaction with bacterial DNA upon entry into the cell. In addition, albofungin’s antibiofilm activity was attributed to its ability to inhibit peptidoglycan biosynthesis, flagellar assembly pathways, and secretion system proteins (Fig. 8). Thus, understanding the mechanism behind albofungin is essential for its further development as a potential antibacterial and antibiofilm agent.

## MATERIALS AND METHODS

### Materials

3,3′-Dipropylthiadicarbocyanine iodide (DiSC_3_(5)), 3-(4,5-dimethylthiazol-2-yl)-2,5-diphenyltetrazolium bromide (MTT), Triton X-100, DMSO, glutaraldehyde solution, and lipopolysaccharides (LPSs) from *E. coli* O111:B4 were purchased from Sigma-Aldrich (Burlington, USA). DAPI, FM 4-64 dye, SYTOX green nucleic acid stain, SYTO 9 green fluorescent nucleic acid stain, phosphate-buffered saline (PBS) tablets, and ProLong glass antifade mountant were purchased from Thermo Fisher (Waltham, USA). The purified albofungin was further conjugated with FITC by a TGpeptide company (Jiangsu, China). *Vibrio alginolyticus* 1597 and *V. parahaemolyticus* 1482 strains were given by Prof. Chen Sheng from the City University of Hong Kong.

### Isolation and purification of albofungins

Herein, albofungin and its derivatives were isolated and purified (18). The culture of *S. chrestomyceticus* BCC 24770 was extracted thrice with an equal volume of ethyl acetate, followed by evaporation to yield a dried crude extract. The dried crude extract was dissolved in methanol (MeOH) and subjected to fractionation using a C18 silica gel column chromatography. Then, a gradient elution with increasing concentrations of MeOH: H_2_O (20:80–100:0) was employed to obtain various fractions, and further purified by preparative high-performance liquid chromatography (HPLC, Waters 2695, Milford, MA, United States). The present study used HPLC to determine the purity of more than 95% of the compounds, and their structures were determined by the Bruker NMR spectrometers (Bruker, Billerica, MA, United States) (18).

### Time-kill and growth curve kinetics

For the time-kill kinetics, the bacteria were incubated to the early logarithmic growth stage (OD_600_ = 0.1) and treated with compounds with the final concentrations of 4 × MIC and 10 × MIC in the 1.5-mL tubes. Then, 100 µL of samples was taken out at each time point, resuspended into sterile PBS, and subsequently plated onto LB agar plates with a series of dilutions. Afterward, the number of colonies on the plates was counted after overnight incubation at 37°C.

For the growth curve kinetics, the bacteria in the early logarithmic phase were treated with compounds with final concentrations of 1 × MIC, 2 × MIC, 4 × MIC, and 8 × MIC in 96-well plates. Then, the plate was incubated at 37°C for 6 h, and the OD_600_ measurements were taken by CLARIOstar Plus microplate reader (BMG LABTECH, Hesse, Germany) every 5 min. Albofungin and its derivatives’ MIC values against multi-drug-resistant *Vibrio* strains were tested as described previously (18).

### Hemolysis assay

The red blood cells from a healthy rabbit were taken. Afterward, compounds were diluted with sterile PBS to different concentrations and then incubated with 2% blood cells (diluted in PBS) at 37°C for 1 h. Then, samples were centrifuged at 10,000 rpm for 5 min, and the supernatants were taken into a 96-well plate. Subsequently, the absorbance was measured at a wavelength of 570  nm by CLARIOstar Plus microplate reader. Afterward, 0.5% Triton X-100 was used as a positive control, whereas 1% DMSO dissolved in PBS was used as the negative control.

### Biofilm inhibition and eradication assay

The overnight culture of *V. parahaemolyticus* cells was diluted into around 10^7^ CFU mL^–1^ in LB medium added with 0.5% of glucose. The biofilm inhibition assay was measured through the MTT assay (20). The absorbance was determined using the CLARIOstar Plus microplate reader under 570 nm. For the biofilm eradication assay, the preformed biofilms (24 h) were washed twice in sterile PBS to remove the planktonic bacteria. Herein, compounds were serially diluted in an LB medium (with 0.5% glucose) and subsequently added to the 96-well plates and incubated at 37°C for 24 h. Finally, as described above, biofilms were quantified for the biofilm inhibition assay.

### Swarming mobility

*V. parahaemolyticus* cells were grown into a logarithmic phase (OD_600_ = 0.6) and treated with 0, 0.03, and 0.06 µg mL^−1^ albofungin. Then, 10 µL of samples was promptly inoculated at the center of the surface of an LB broth media with 0.5% agar plates. Finally, we measured the diameters of the bacterial swarming after 24 hr.

### DNA interaction assay

The TIANamp Genomic DNA Kit (Tiangen, Beijing, China) was used to extract the genomic DNA of the *V. parahaemolyticus* and mixed it with an equal volume of albofungin solutions. The final concentrations of albofungin were set as 0, 3, 6, 12, 24, and 48 µg mL^−1^. Then, DNA shift was measured by 1% agarose gel electrophoresis after incubating for 30 min at 37°C.

### LPS-binding study

ITC measurements for the LPS-binding activity of albofungin were performed at 37°C using a Malvern MicroCal PEAQ-ITC (Malvern Instruments, Malvern, United Kingdom). Then, 25 µM LPS and 50 µM albofungin solutions were prepared in a PBS buffer (pH 7.4). After thermal equilibration, 2 µL aliquots of the albofungin solution were added every 5 min into the LPS solution at 37°C with constant stirring. Finally, data were fitted to a one-site-binding model using MicroCal PEAQ-ITC Analysis software.

### Scanning electron microscopy observation

The bacteria were inoculated in an LB medium and grown until the logarithmic phase was reached (OD_600_ = 0.6). They were treated with 0.03 µg mL^−1^ (1 × MIC) of albofungin for 20 min at 37°C. For the biofilm samples, the biofilm was formed on a glass coverslip in 24 well plates for 24 h and treated with 0.12 µg mL^−1^ of albofungin for 24 h at 37°C. Afterward, the bacteria and biofilm samples were collected and washed in the sterile PBS to remove the compound and then fixed in the 2.5% glutaraldehyde in PBS at 4°C overnight. Then, the samples were dehydrated in a sequence of increasing ethanol concentrations (30%, 50%, 70%, 90%, and 100%), followed by an air dry. Finally, the dried samples were gold-coated and observed using SEM (JSM-6390, JEOL, Akishima, Tokyo, Japan).

### Bacterial cell membrane permeability assay

The *V. parahaemolyticus* cells in the logarithmic phase were adjusted to an OD_600_ = 0.2 and washed with sterile PBS. For the DiSC_3_(5) assay, to achieve equilibrium in K+ concentration, 0.1 M potassium chloride was added and incubated for 30 min. Then, 1 µM DiSC_3_(5) was added into the bacterial suspension for 1 h of incubation. In addition, SYTOX green dye was added to the bacterial suspension and incubated for 15 min for the SYTOX green assay. Then, the bacterial suspension and compounds with different concentrations were transferred to a black, clear-bottom 96-well black plate at 100 µL per well, and the fluorescence uptake was immediately monitored using CLARIOstar Plus Microplate Reader with excitation and emission wavelengths of 622  and 670 nm for the DiSC_3_(5) or excitation and emission wavelengths of 485  and 520 nm for the SYTOX green, respectively. Herein, polymyxin B-treated cells were used as the positive control.

### Fluorescence microscopy

*V. parahaemolyticus* cells were grown in an LB medium, and the logarithmic phase was achieved (OD_600_ = 0.6). Then, the FITC-albofungin compound was added into the bacterial suspensions and incubated for 20 min, and DAPI (1 µg mL^−1^) and FM4-64 (1 µg mL^−1^) were added and incubated at room temperature for 10 min. Cells were collected by centrifuge, washed, and resuspended in sterile PBS (100 µL). Finally, 2 µL of the cells was then placed on a precleaned slide and mounted with ProLong glass antifade mountant. A cover glass (0.13 mm thick) was carefully placed over the cells.

For the biofilm samples, the biofilm was grown on the glass slides at the bottom of 24-well microplates for 24 h at 37°C and treated with albofungin for 20 h. Then, the coverslips were washed twice in PBS and stained with SYTO 9 green fluorescent nucleic acid stain for 15 min. Samples were washed and subsequently imaged by LSM 980 confocal microscope (Zeiss, Oberkochen, Germany).

### Sample preparation for proteomics

Bacteria were grown to the logarithmic phase (OD_600_ = 0.4) and treated with albofungin (0.03 µg mL^−1^) for 0.5 h. Meanwhile, the preformed biofilms (24 h) in a 12-well plate (Corning, New York, USA) were rinsed with sterile PBS and treated with albofungin (0.06 µg mL^−1^) for 1 h. Then, planktonic bacteria and biofilm cells that were not treated with albofungin were collected as controls. All samples were subjected to three biological replicates. After collecting the samples, the cell pellet was suspended in 300 µL of lysis buffer consisting of 8 M urea and 50 mM Tris-HCl at a pH of 8.0 and sonicated for 5 min. Protein concentration was measured using the Bradford protein assay (Bio-Rad, Hercules, USA). After protein quantification, samples were further precipitated using four volumes of cool acetone overnight and centrifuged at 15,000 × *g* for 30 min at 4°C. The PreOmics iST-PreOn Kit (PreOmics, Planegg, Germany) was used to digest and purify the dried pellet on the automated PreON machine (PreOmics, Planegg, Germany).

The purified sample was reconstituted in 0.1% formic acid in water with a final concentration of 200 ng µL^−1^, and 1 µL was injected into Bruker nanoElute ultrahigh-performance liquid chromatograph (UHPLC, Bruker Daltonics, Bremen, Germany) and separated on an IonOpticks 25 cm Aurora series emitter column with CaptiveSpray insert (25 cm × 75 µm internal diameter, 120 Å pore size, 1.6 µm particle size C18) at a ﬂow rate of 0.3 µL min^−1^, from 2% to 95% acetonitrile for 30 min. Using positive electrospray mode, we obtained mass spectra in the range of *m*/*z* 100–1,700 using the Bruker TimsTOF Pro mass spectrometer (Bruker Headquarters in Billerica, MA, United States). As reported previously (37), the collision energy was increased linearly from 27 to 45 eV with increasing ion mobility, while the ion mobility scan range was set between 0.85 and 1.30 Vs/cm^2^.

### Database searching and label-free quantification of proteomics data

In addition, the Transdecoder v5.5.0 was used to convert the genome sequence of *V. parahaemolyticus* into a protein database (38). The proteins were annotated by performing a BLASTp search (v2.7.1) against the protein database of *V. parahaemolyticus* available on NCBI. Afterward, data were converted into MGF format files and processed database search using the PEAKS Studio software version X pro. Subsequently, the parameters were configured with a parent mass error tolerance of 15.0 ppm, a fragment mass error tolerance of 0.05 Da, and a monoisotopic type. Only proteins and peptides with false discovery rates (FDR) of <1% were filtered out. Proteins were considered differentially expressed if their fold change was greater or less than twofold and permutation-based FDR was less than or equal to 0.05. Statistical tests were performed using Student’s *t*-test calculations.

Furthermore, the Kyoto Encyclopedia of Genes and Genomes (KEGG) database through the KEGG Automatic Annotation Server (KAAS), the Gene Ontology (GO) through OmicsBox version 1.4.11 (BioBam, Valencia, Spain), and the KOG through eggNOG-mapper v2 were used to annotate the protein sequence database (39). For the enrichment analysis, the KEGG and GO pathways were enriched through cumulative hypergeometric distribution by the OmicShare online tool (https://www.omicshare.com/).

### qPCR analysis

Herein, samples were prepared in the same way as described for proteomics. The RNeasy Mini Kit (QIAGEN, Hilden, Germany) was used to extract RNA based on the manufacturer’s protocols. Then, the genomic DNA was removed, and cDNA was synthesized by HiScript III All-in-one RT SuperMix Perfect for qPCR Kit (Vazyme, Nanjing, China). Moreover, qPCR was carried out using LightCycler 480 SYBR Green I Master (Roche, Basel, Switzerland) on a Roche Diagnostics LightCycler 480 Instrument II Real-time PCR System machine. Subsequently, the glyceraldehyde-3-phosphate dehydrogenase gene was utilized as an internal control to normalize the relative expression levels. The 2^–ΔΔCT^ method was used to calculate the relative fold changes in the expression level of each gene (40). Supplementary Table 1 lists all the primers used in the present study.

### Statistical analysis

The significant differences in the data were analyzed using one-way ANOVA, and standard deviation was calculated using GraphPad Prism 9. All experiments were carried out in triplicate.

## Data Availability

The mass spectrometry proteomics data have been deposited with ProteomeXchange via the PRIDE partner repository with the dataset identifier PXD040329.

## SUPPLEMENTAL MATERIAL

Supplemental Fig. S1 to S6, Table S1 spectrum.01563-23-s0001.docx Supplemental information.

ASM does not own the copyrights to Supplemental Material that may be linked to, or accessed through, an article. The authors have granted ASM a non-exclusive, world-wide license to publish the Supplemental Material files. Please contact the corresponding author directly for reuse.
