# Supplementary material for: Mode of action of antimicrobial agents albofungins in eradicating penicillin- and cephalosporin-resistant Vibrio parahaemolyticus biofilm
Source: Microbiol Spectr. 2023 Aug 23;11(5):e01563-23. doi: 10.1128/spectrum.01563-23 (PMC10581126; doi:10.1128/spectrum.01563-23)
Supplement: Supplemental Fig. S1 to S6, Table S1 — Supplemental information. [file spectrum.01563-23-s0001.docx]

**Supplementary information**

Mode of action of antimicrobial agents albofungins in eradicating penicillins-and-cephalosporins-resistant *Vibrio parahaemolyticus* biofilm

Weiyi She^a,b,†^, Aifang Cheng^a,b,†,#^, Wenkang Ye^a,b^, Ping Zeng^c^, Hao Wang^a,b^, and Pei-Yuan Qian^a,b,#^

^a^Southern Marine Science and Engineering Guangdong Laboratory (Guangzhou), Guangdong 511458, China

^b^Department of Ocean Science, Hong Kong University of Science and Technology, Hong Kong, China

^c^School of Pharmacy, Faculty of Medicine, The Chinese University of Hong Kong, Hong Kong, China

^#^Address correspondence to Pei-Yuan Qian, [boqianpy@ust.hk](mailto:boqianpy@ust.hk), and Aifang Cheng [chengaf@ust.hk](mailto:chengaf@ust.hk).

^†^These authors contributed equally to this work.

**Content:**

**Table S1**. Primers used in the qPCR analysis.

**Figure S1**. (A) ^1^H NMR spectrum of albofungin (**1**) in DMSO-*d*_6_. (B) ^1^H NMR spectrum of albofungin A (**2**) in DMSO-*d*_6_. (C) ^1^H NMR spectrum of chloroalbofungin (**3**) in DMSO-*d*_6_. (D) ^1^H NMR spectrum of chrestoxanthone A (**4**) in DMSO-*d*_6_. (E) ^1^H NMR spectrum of chrestoxanthone C (**5**) in DMSO-*d*_6_.

**Figure S2**. Hemolytic analysis of compounds against rabbit red blood cells. (A) compound **5** (B) compound **1** (C) compound **2**.

**Figure S3**. Principal-component analysis (PCA) of biofilm control (group A) and planktonic bacteria control (group B) samples before albofungin treatment.

**Figure S4**. Gene ontology (GO) enrichment analysis of the affected processes in planktonic bacteria (E) and (F) biofilm after albofungin treatment.

**Figure S5**. Interaction between albofungin and genomic DNA of *Vibrio parahaemolyticus* by DNA gel retardation analysis.

**Figure S6**. ITC assay for the detection of interaction between albofungin and LPS. The Upper represents albofungin added into LPS, and the lower represented ddWater added into LPS.

**Table S1**. Primers used in the qPCR analysis.

| **Primer name** | **Sequence(5’-3’)** |
| --- | --- |
| cheB_F | CGTTGCGGTAAATGGCAGAG |
| cheB_R | CCCATCATGAGTCAGGGACG |
| fliH_F | CTGGGTAAAGCAGAAGGCGT |
| fliH_R | TTGAACCTCGACATGCACCA |
| fliM_F | GGCGTCGAGTTCGAATACCT |
| fliM_R | CTCGACCATGGAATACGGCA |
| cheW_F | AACAGCGTACTTTCCAGCGA |
| cheW_R | AGCGAGCAAGTCAAGCTCAT |
| dnaN_F | CCGACCAACCCTGCCTATTT |
| dnaN_R | AACTTACGCGAAGGAACGGT |
| mpl_F | ACGGGTTCTGATGCCAATGT |
| mpl_R | TCGCGTTACCAATCACCACA |
| MurE_F | ATGGGCACCACAGGTAATGG |
| MurE_R  MurQ_F | GCCAAAGAAGCCAGCGTATG  AGGACAAACTCGTTCCGCTT |
| MurQ_R | GCATTCCGAGGCATCCAGTA |
| MurA_F | GTCGATACGGCGGATTTCCT |
| MurA_R | GTCTGCTACGACACTGTGCT |

**
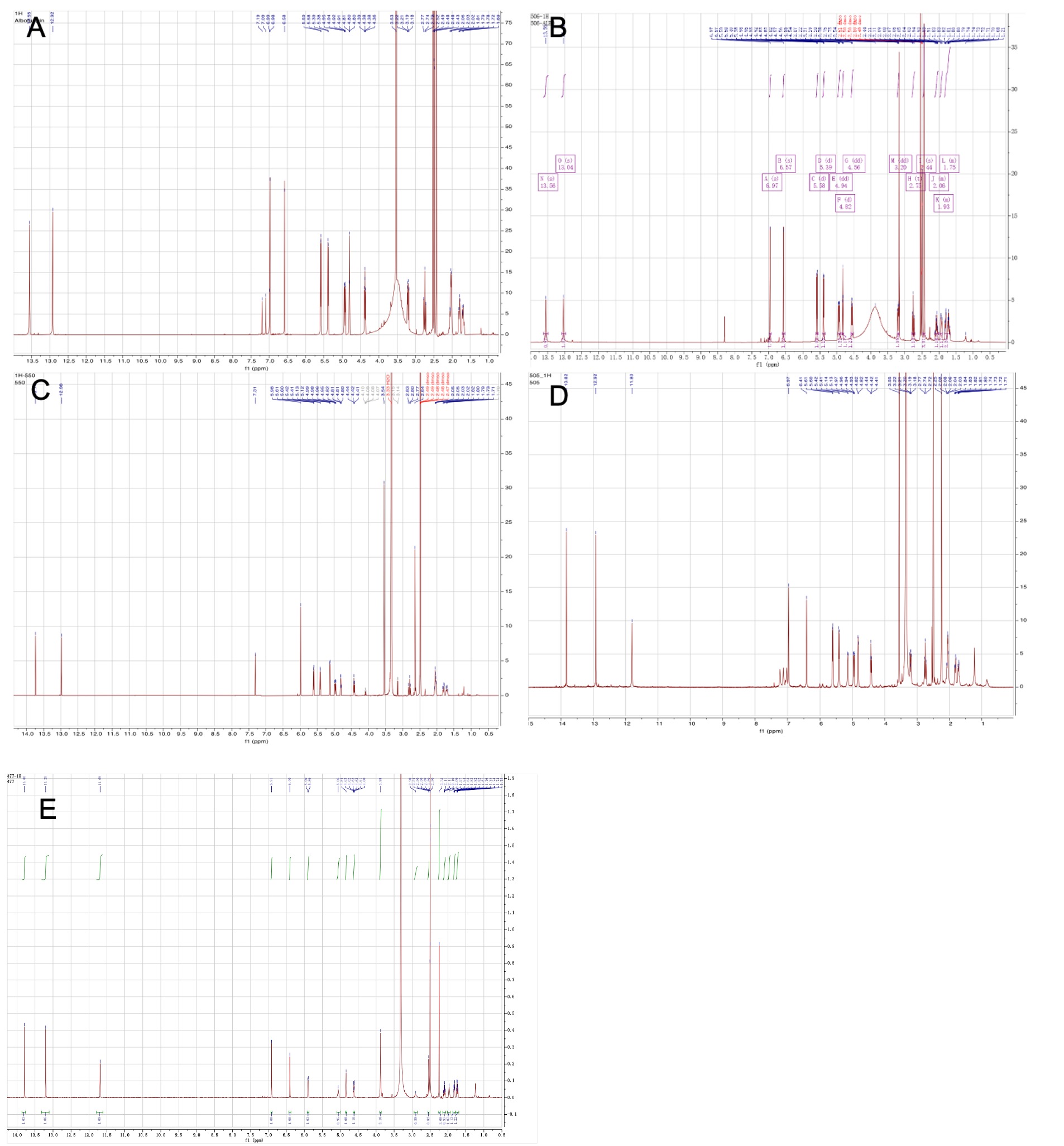
**

**Figure S1**. (A) ^1^H NMR spectrum of albofungin (**1**) in DMSO-*d*_6_. (B) ^1^H NMR spectrum of albofungin A (**2**) in DMSO-*d*_6_. (C) ^1^H NMR spectrum of chloroalbofungin (**3**) in DMSO-*d*_6_. (D) ^1^H NMR spectrum of chrestoxanthone A (**4**) in DMSO-*d*_6_. (E) ^1^H NMR spectrum of chrestoxanthone C (**5**) in DMSO-*d*_6_.

**
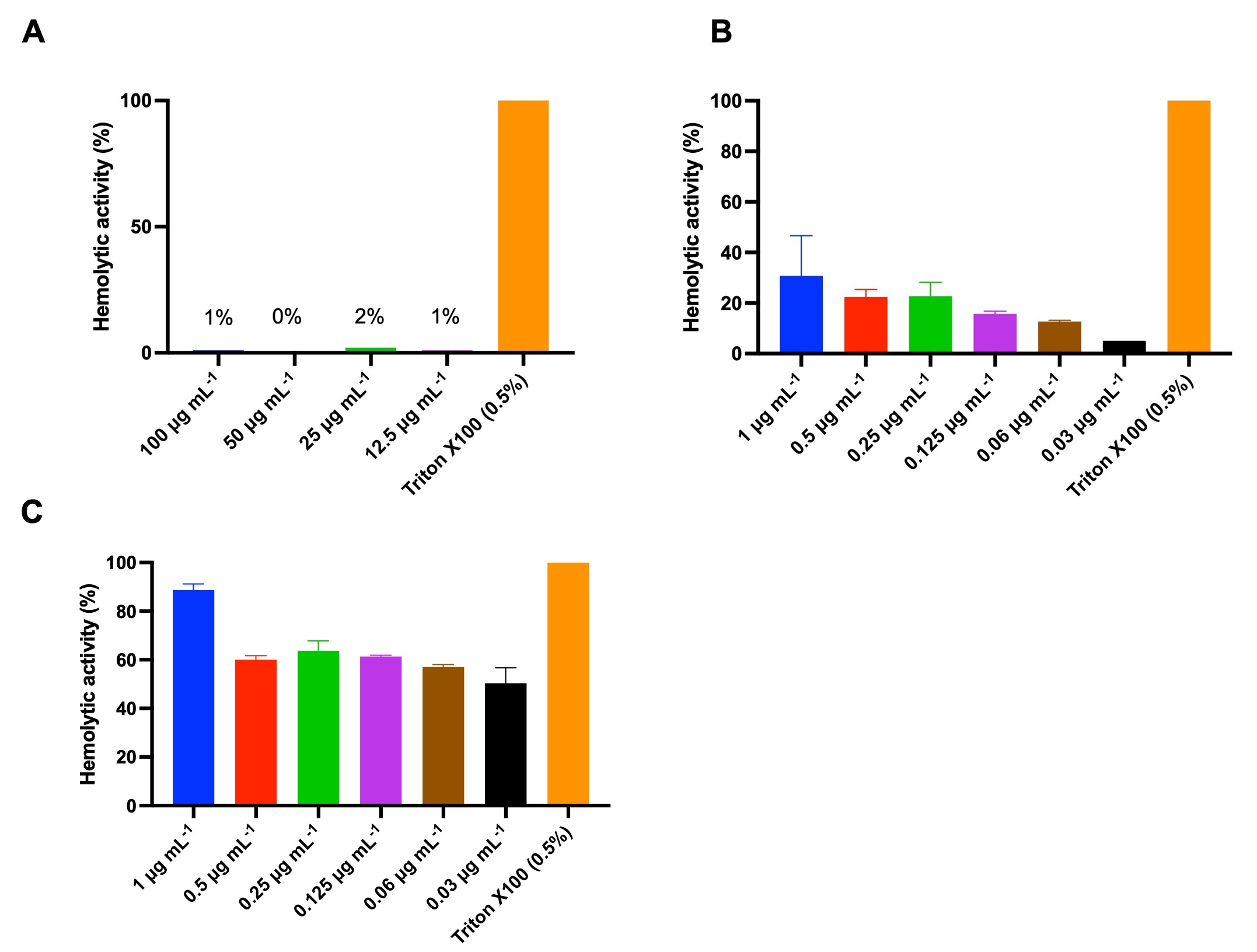
**

**Figure S2**. Hemolytic analysis of compounds against rabbit red blood cells. (A) compound **5** (B) compound **1** (C) compound **2**.


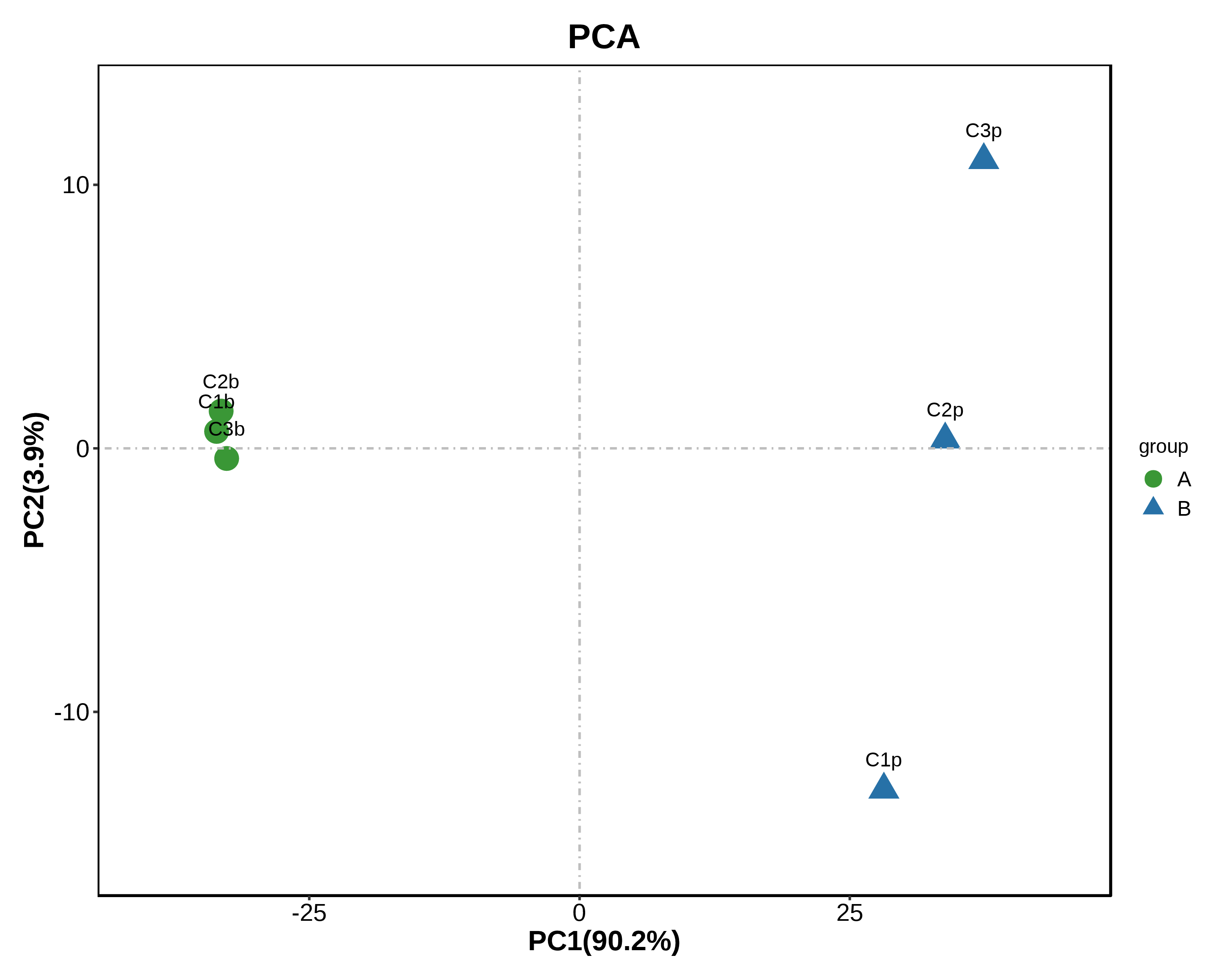


**Figure S3**. Principal-component analysis (PCA) of biofilm control (group A) and planktonic bacteria control (group B) samples before albofungin treatment.


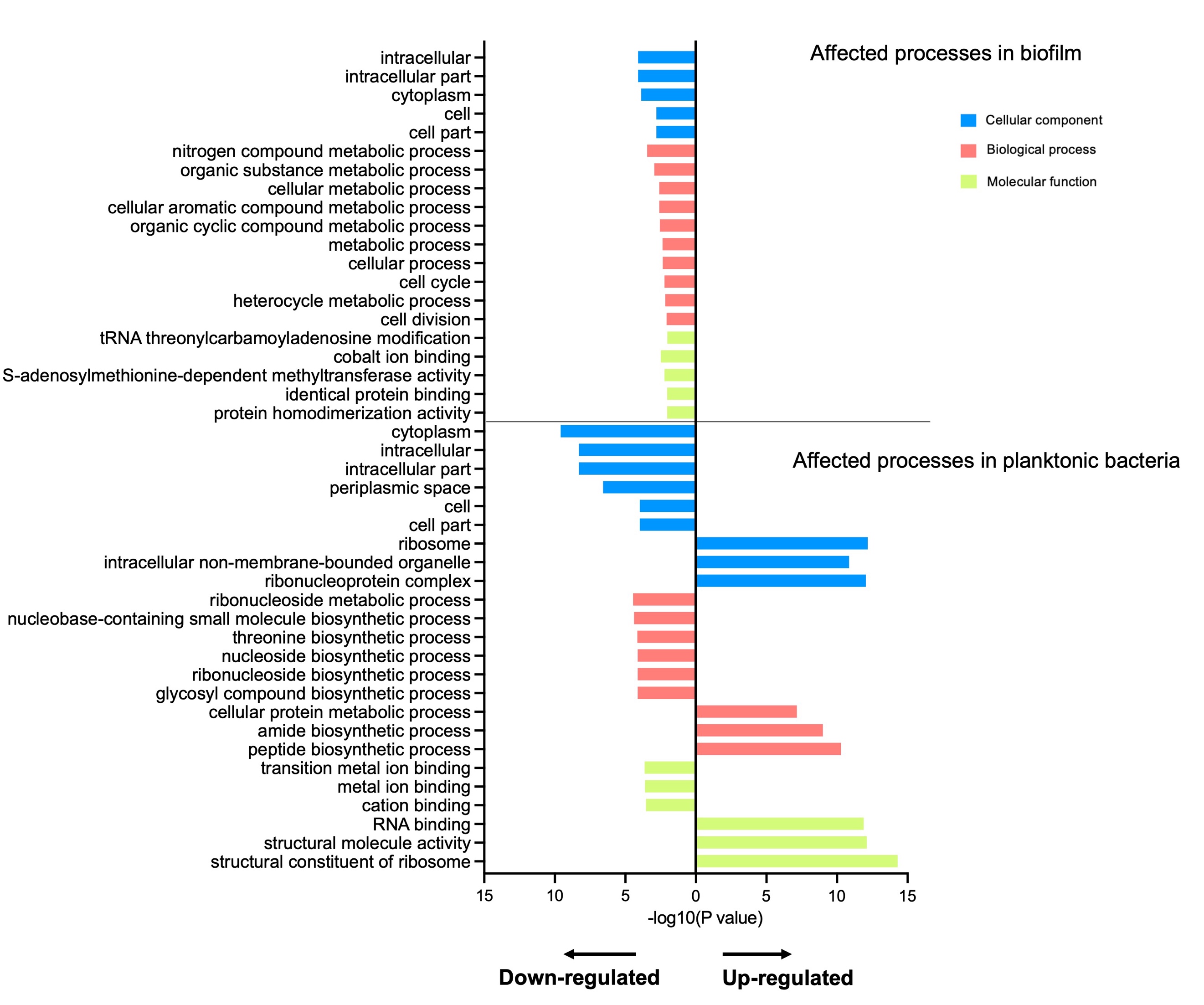


**Figure S4**. Gene ontology (GO) enrichment analysis of the affected processes in planktonic bacteria (E) and (F) biofilm after albofungin treatment.


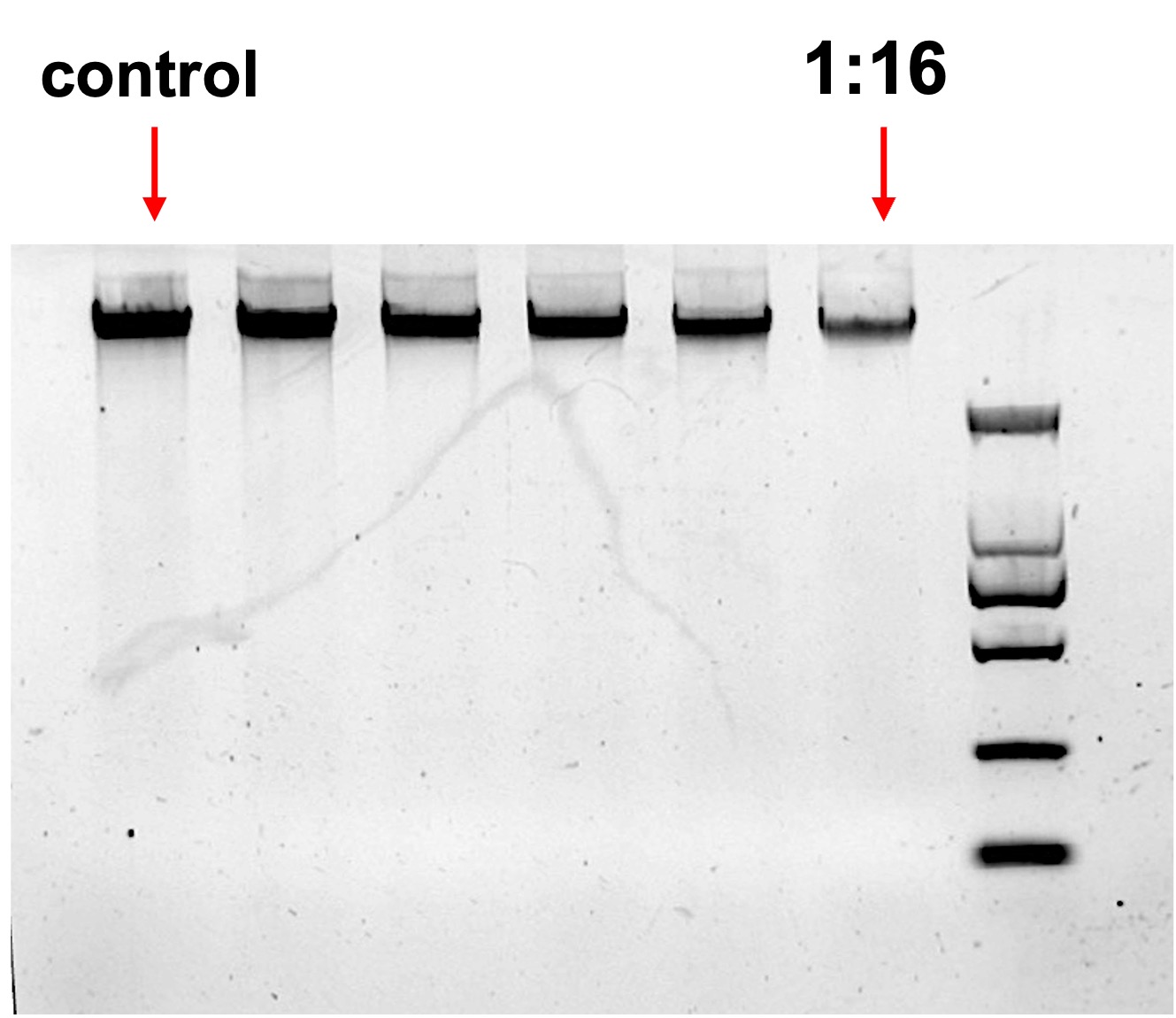


**Figure S5**. Interaction between albofungin and genomic DNA of *Vibrio parahaemolyticus* by DNA gel retardation analysis.

**
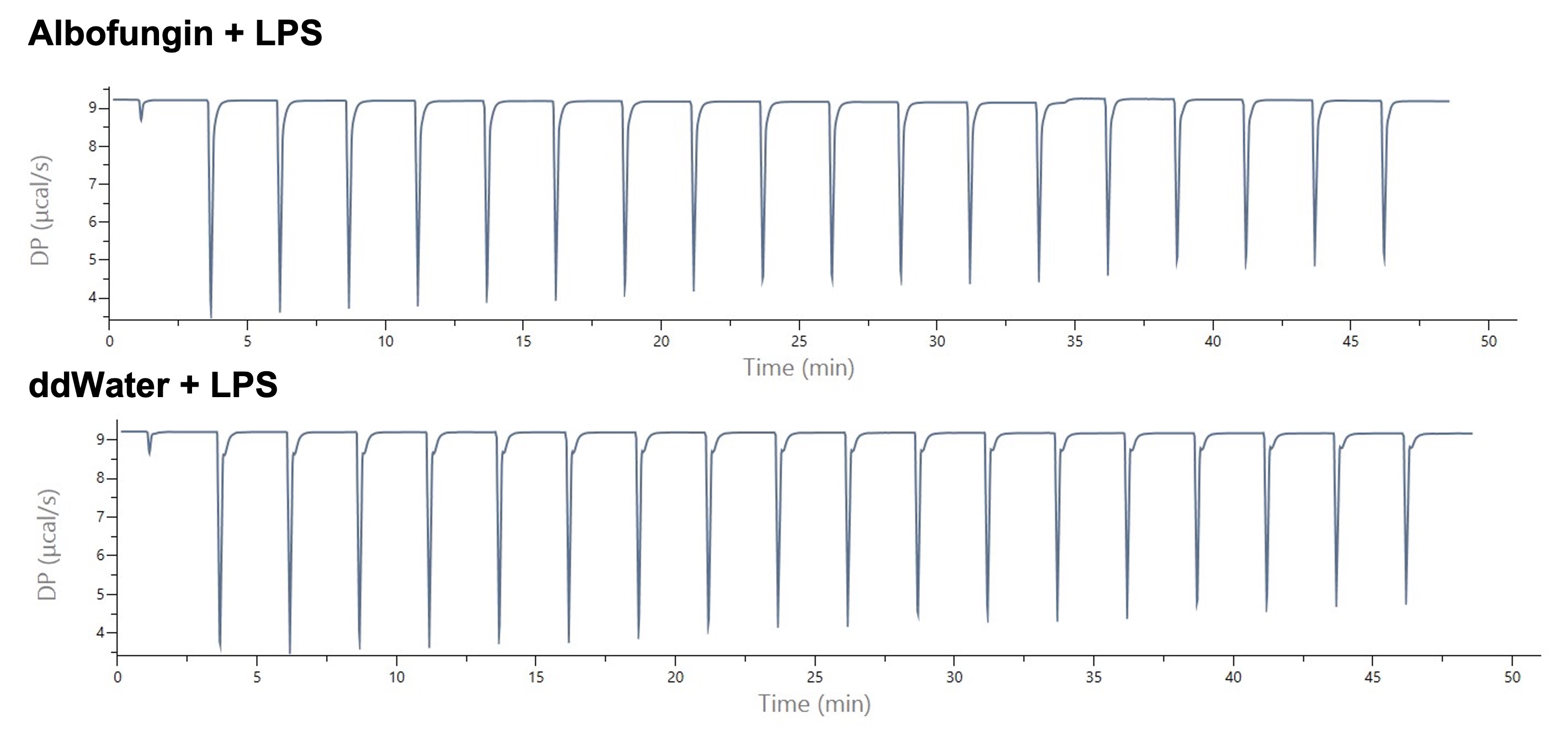
**

**Figure S6**. ITC assay for the detection of interaction between albofungin and LPS. The Upper represents albofungin added into LPS, and the lower represented ddWater added into LPS.
